# Spatio-Temporal Gap Filling of Sentinel-2 NDI45 Data Using a Variance-Weighted Kalman Filter and LSTM Ensemble

**DOI:** 10.3390/s25175299

**Published:** 2025-08-26

**Authors:** Ionel Haidu, Zsolt Magyari-Sáska, Attila Magyari-Sáska

**Affiliations:** 1 LOTERR (Laboratoire des Observations de Territoires), Université de Lorraine, F-57000 Metz, France; ionel.haidu@univ-lorraine.fr; 2 Gheorgheni Extension, Faculty of Geography, Babes-Bolyai University, 535500 Gheorgheni, Romania; 3 Faculty of Mathematics and Computer Science, Babeș-Bolyai University, 400084 Cluj-Napoca, Romania; attila.magyari@stud.ubbcluj.ro

**Keywords:** ensemble model, gap filling, spatio-temporal analysis, model uncertainty, vegetation index

## Abstract

This study aims to reconstruct NDI45 missing values due to cloud cover while outlining the importance of vegetation health for the climate–carbon cycle and the benefits of the NDI45 index for high canopy area indices. The methods include a novel hybrid framework that combines a deterministic Kalman filter (KF) and a clustering-based LSTM network to generate gap-free NDI45 series with 20 m spatial and 5-day temporal resolution. The innovation of the applied method relies on achieving a single-sensor workflow, provides a pixel-level uncertainty map, and minimizes LSTM overfitting through clustering based on a correlation threshold. In the northern Pampas (South America), this hybrid approach reduces the MAE by 22–35% on average and narrows the 95% confidence interval by 25–40% compared to the Kalman filter or LSTM alone. The three-dimensional spatio-temporal analysis demonstrates that the KF–LSTM hybrid provides better spatial homogeneity and reliability across the entire study area. The proposed framework can generate gap-free, high-resolution NDI45 time series with quantified uncertainties, enabling more reliable detection of vegetation stress, yield fluctuations, and long-term resilience trends. These capabilities make the method directly applicable to operational drought monitoring, crop insurance modeling, and climate risk assessment in agricultural systems, particularly in regions prone to frequent cloud cover. The framework can be further extended by including radar backscatter and multi-model ensembles, thus providing a promising basis for the reconstruction of global, high-resolution vegetation time series.

## 1. Introduction

The health of Earth’s vegetation directly shapes climate–carbon cycle feedbacks, regional water balances, and global food security, thus affecting at least four UN Sustainable Development Goals (SDGs 2, 6, 13, 15) [1,2]. Remote sensing provides scale-independent time series enabling drought early warning services and climate-smart insurance and restoration programs [3,4]. However, there is growing evidence that vegetation resilience is sharply reduced in water-scarce regions, increasing the risk of failure in key biomes such as the Amazon and boreal forests [5]. Reliable, cloud-independent temporal and spatial coverage is therefore not just a convenience, but a prerequisite for risk management. The importance of this study lies in addressing the above-mentioned critical need for reliable, cloud-independent vegetation monitoring capable of supporting both global and local sustainability goals. By focusing on high-leaf-area-index (LAI) agroecosystems, this research offers a methodology that produces gap-free vegetation index time series with quantified uncertainties.

The Normalized Difference Vegetation Index (NDVI) has been used as a fast, scale-independent indicator of “greenness” for nearly half a century. Its simplicity is also its main weakness: the red band falls entirely on the absorption maximum of chlorophyll-a, so the signal is predominantly confined to the sunlit leaf surface [6]. As the leaf area index (LAI) exceeds ~3 m^2^ m^−2^, red absorption saturates, the NDVI curve flattens, and excess biomass, nitrogen, or water content is hidden [7,8]. In the past decade, several modified indices—SAVI [9], EVI/EVI2 [10]—and various narrowband variants [11,12] have been developed to alleviate these saturation and background sensitivity problems, but significant uncertainty still remains in the high-LAI range. Recent research, based on Sentinel-2 and UAV images, has also shown that these indices have a close relationship with above-tree biomass and carbon sequestration capacity [13,14]. A study reviewing 1986–2021 showed that deep learning-based time series reconstructions outperform traditional smoothing-interpolation methods in cloudy tropical and boreal regions, but further research is needed to explore the parameter sensitivity and regional applicability of the methods [15].

In this study, we use the Normalized Difference Index based on Sentinel-2 bands 4 and 5 (NDI45). Compared to the traditional NDVI, which uses a red–NIR pair, NDI45 leverages the red-edge region where chlorophyll absorption begins to decline. This reduces the saturation effects that limit NDVI sensitivity at high leaf area index (LAI) values (>3 m^2^ m^−2^) and allows for a more linear relationship with canopy cover up to LAI ≈ 6 [16].

The NDI45 index, calculated from Sentinel-2 B4 (665 nm) and B5 (705 nm) bands, has been extensively validated across diverse contexts. Field and multi-biome studies have shown that NDI45 explains substantially more variance in above-ground biomass and canopy chlorophyll content than NDVI, especially in dense or mature vegetation. In Java, NDI45 over private forests explained 72% of the above-ground biomass, outperforming six conventional indices [17]. In the temperate forests of northern China, it improved the ML estimate of canopy cover by 15% [18], while in irrigated cotton, it showed the strongest relationship between stem and water potential [19]. Laboratory spectral tests indicated a six-fold gain in pigment sensitivity compared to the classic red–NIR pair [20]. Based on this evidence, NDI45 is referred to in the literature as a “sweet-spot” index, combining the pigment sensitivity of the red band with the desaturation of the red edge.

**Figure 1:**
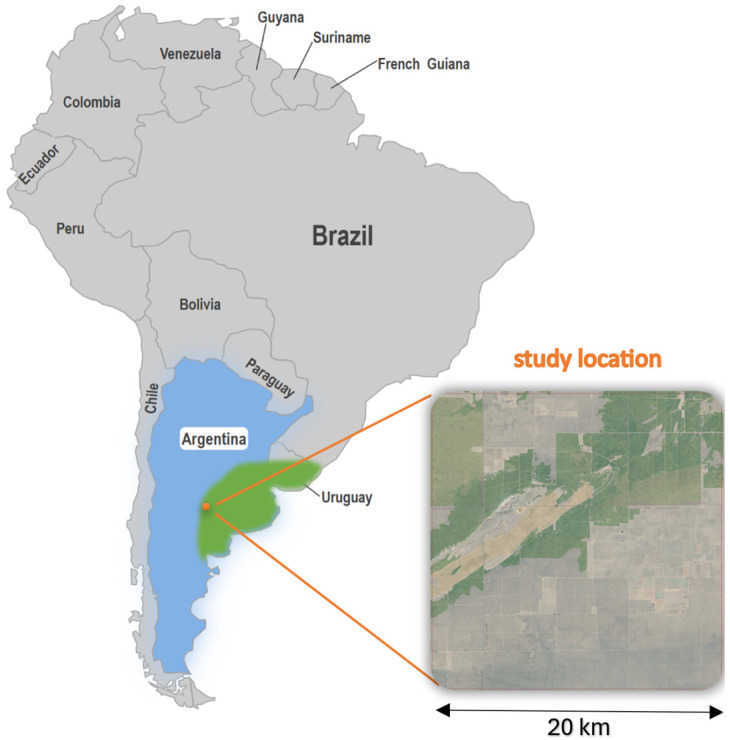
Study area (Argentina, Pampas).

The selected study area is located in the humid–arid transition zone, on the western edge of the Pampas (Figure 1). Annual precipitation varies between 600 and 850 mm, with a strong ENSO dependence; in the 2017/18 La Niña year, yields in the region fell by 30% [21,22]. Sentinel-2 returns every five days, but the humid periods and cumulus cloud formation in the Pampas make up to 30–40% of the images unusable. Missing observations distort stress diagnosis, weaken biomass and yield models, and degrade regional early- warning systems [3]. A continuous, cloud-independent NDI45 time series is therefore essential for reliable monitoring of sustainability indicators [23] and vegetation resilience trends [5].

Existing gap-filling methods for vegetation indices typically rely on three main strategies: (1) state–space models, which are physically interpretable but struggle with strong non-linearities; (2) classical smoothing/interpolation (e.g., Whittaker, Savitzky–Golay), which can underfit rapid phenological changes and lack uncertainty estimates; and (3) machine/deep learning approaches, which capture complex spatio-temporal patterns but require dense training data and rarely provide pixel-level reliability.

A variety of gap-filling techniques have been proposed for vegetation index time series, particularly for NDVI, to mitigate the effects of cloud contamination. Logistic fitting captures seasonal patterns with few parameters but oversimplifies abrupt changes. HANTS and harmonic methods reduce noise and reconstruct periodic signals but can underfit rapid transitions and are sensitive to parameter choice, especially in heterogeneous areas. As Julien and Sobrino [24] note, parameter optimization is critical, and no single method works best under all conditions.

The objectives of this study are (1) to develop a hybrid variance-weighted Kalman filter–LSTM framework for reconstructing cloud-affected NDI45 time series and (2) to quantify the accuracy and uncertainty of the reconstruction at pixel level and apply it to a test area in the northern Pampas study area of Argentina. The methodological novelty of this study lies in the integration of a deterministic Kalman filter (KF) with a long short-term memory (LSTM) deep learning network through a variance-weighted fusion scheme. In our framework, KF leverages explicit state–space dynamics to produce physically interpretable and statistically optimal short-gap predictions, while LSTM captures nonlinear, long-term temporal patterns and spatial similarities supported by previous indices (NDVI, EVI, ReCI) [25]. The two outputs are then combined at each pixel and time step using weights inversely proportional to their forecast variances, so that the more certain model contributes more strongly to the final estimate. To the authors’ knowledge, this is the first application of this combination to a red-edge-based index in Pampas. The study area was not selected based on any specific climatic or other characteristics of the region, but specifically to validate the performance of the proposed data augmentation methodology on a real, large-scale Sentinel-2 time series.

## 2. Materials and Methods

### 2.1. Data

For the research, we used Sentinel-2 L2A surface reflectance products provided by the Copernicus programme (European Space Agency, Paris, France) from the period 25 March 2019–31 December 2024. Based on the tile identifier (20 HLE) for our study area, we primarily used the download request command for AWS (Amazon Web Services) to acquire the available imagery.

We ensured that all downloaded images had been processed or reprocessed with the Processing Baseline (PB) 05.00 algorithm (“Collection-1”), so that the band values were calculated using the same procedure at all times, which is a basic requirement for long-term time series analyses.

The Collection-1 reprocessing unified the Sentinel-2A/B archive with improved GRI geometry, a 30 m DEM base, and harmonized radiometry [26]. The different offsets and cloud masks of the previous 02.xx–04.xx versions cause temporal incoherence; PB05.xx was created to eliminate this [27]. By reprocessing, the archive raw data were also brought to the surface reflectance level with the same pipeline [28].

Considering the five-day return rate of the Sentinel-2A and -2B satellite pair, after downloading from AWS and checking the file list, it turned out that imagery for some time points was missing; this was downloaded—where available—from the Copernicus Data Space Browser interface. Finally, a five-day time series with 422 elements was compiled for all 1,019,000 grid points (1019 × 1000 cells) of the study area, with only the date, 17 August 2023, remaining as a gap in each time layer.

Although Sentinel-2 L2A products also include 10, 20, and 60 m pixels, we chose the 20 m resolution because only at this level is the Scene Classification Layer (SCL) available—an 11-class map that classifies each pixel. According to ESA’s official validation, SCL has an average accuracy of 91.5% for clear surfaces and 94.8% for clouds. Recent improvements have further increased the reliability of cloud and shadow filtering, especially for topographic shadows and snow misidentifications [29], making SCL-based masking play an important role in machine learning processing chains and significantly increase the clean pixel ratio [30].

### 2.2. Preprocessing

Considering the entire image set, the NDI45 index was calculated from the 20 m resolution B4 (665 nm) and B5 (705 nm) bands (Equation (1)).

(1) $$NDI45=\frac{B5-B4}{B5+B4}$$

Using the SCL codes, we replaced all pixels classified as no-data, saturated/defective, cloud, cloud shadow, or cirrus with NA. The study area clipping, band extraction, NDI45 calculation, and quality masking were all performed using R-based scripts, resulting in a NetCDF data file containing the 422-element NDI45 time series for each cell.

**Figure 2:**
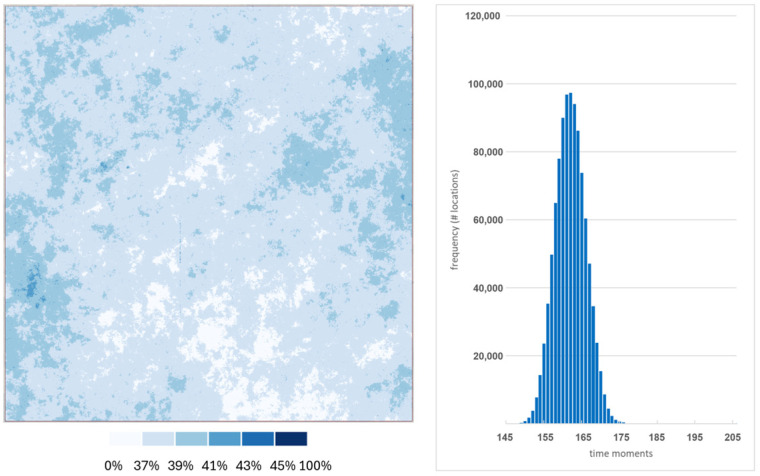
Spatial distribution and histogram of cloud-free percentage.

The spatial distribution of missingness reveals that there are no locations with full data cover, and the majority of locations are covered by data on 34–49% of dates (Figure 2). The histogram for the spatial distribution of cloud-free locations is bell-shaped with a median of 161 and a mean of 161.9 (stdev = 4). Over 99.7% of locations have between 148 and 175 valid dates (41.9%) representing a cloud-free coverage of 35% and 41.9%, respectively.

### 2.3. Methods

As mentioned in the Introduction, three main methodological approaches have emerged over the last decade to fill the gaps in vegetation indices (mainly NDVI/NDI-type series): (1) classical state–space models, (2) statistical or deterministic smoothing/interpolation methods, and (3) machine learning and deep learning-based predictors. The hybrid KF + LSTM framework proposed in this study actually combines the strengths of these three approaches, and is therefore worth briefly comparing with the literature.

1. State–space-based approaches. Several works use only the Kalman filter or its variants. Sedano and his colleagues [31] generated Landsat-NDVI series in a trend and seasonality model, at a resolution of 30 m/16 days. Moreno-Martínez [32] implemented a bias-aware Kalman filter in the HISTARFM algorithm, which explicitly takes into account the temporal autocorrelation of measurement and model errors. Based on these experiences, in the present study, we also added a non-zero mean, time-series-dependent term to the process-noise covariance to mitigate the bias during longer cloud blocks. The advantage of KF is its physical interpretability and real-time updateability, but the disadvantage is that nonlinear phenomena (e.g., sudden peaks) are difficult to describe with linear dynamics. The current hybrid method retains the deterministic, variance-driven imputation of KF, but supplements it with a nonlinear learning modulus.
2. Smoothing and interpolation techniques. Whittaker or Savitzky–Golay smoothing is still the most common in MODIS products; for example, EGF-WS (Enhanced Gap Filling + Whittaker Smoothing) reconstructs the Landsat–Sentinel–MODIS hybrid NDVI series in the GEE framework [33]. It removes high-frequency noise well, but tends to underfit rapid phenological changes, but the formal uncertainty estimation is missing. Julien and Sobrino [24] performed a parameter optimization study on global simulated NDVI profiles and found that compared to harmonic and logistic fitting, HANTS resulted in a smaller average error in cloud-intensive areas, while IDR was more effective for shorter cloud blocks. Hong et al. [34] introduced OMPEA, an optimized phenology extraction approach combining Savitzky–Golay-based gap filling with spatiotemporal fusion (ESTARFM) to generate daily, 30 m NDVI, applied to mangrove ecosystems.
3. Machine learning and deep learning. The “pure ML” line is represented by several articles: MICE + MLP/KNN combinations for MODIS-NDVI [35], CNN-RNN models with Sentinel-2 and Sentinel-1 fusion [36] and general ML-toolbox with spatio-temporal features [37]. These models provide high accuracy, especially at high missingness, but due to their “black box” nature, uncertainty calibration is weak. Complementing optical time series with radar-based measurements has been a prominent research direction in recent years. Using a Multi-output Gaussian Process model, Pipia and his colleagues [38] demonstrated that the Sentinel-1 RVI time series can overcome long (>2 months) cloud gaps in Sentinel-2 LAI estimation. Similarly, Zhao [39] used MCNN-Seq architecture and Garioud [40] with the SenRVM framework and achieved 6-day effective optical resolution (R^2^ > 0.83) for different vegetation categories using CNN/RNN-based regression. SAR-based, but less computationally demanding, “shallow” machine learning solutions also represent a promising alternative: Lasko [41] achieved MAEs of 0.09–0.21 for NDVI and NDWI from C- and L-band SAR texture attributes using random forest and gradient boosting models, while the resource requirements remained a fraction of those of deep learning models. Van Jaarsveld et al. [42] employed a Random Forest regressor to gap-fill and downscale global EVI to 0.01° resolution, demonstrating spatially and temporally consistent vegetation monitoring. This result supports the fact that the ensemble approach can be relevant not only in terms of accuracy, but also in terms of computational efficiency.

Recent developments in deep learning have introduced several architectures specifically designed to handle temporal imputation tasks with missing or irregularly sampled data. One of the most influential among them is GRU-D (Gated Recurrent Unit with Decay), introduced by Che et al. [43], which incorporates decay mechanisms into GRU cells to model both observed and missing patterns in time series. GRU-D has demonstrated strong performance in healthcare and environmental datasets with high missingness. Another significant contribution is the Temporal Fusion Transformer (TFT) developed by Lim et al. [44], which combines recurrent layers with self-attention, variable selection networks, and interpretable gating mechanisms. TFT excels in multivariate time series forecasting and has shown strong benchmark results across a range of datasets. However, its architectural complexity and computational demands may limit applicability in large-scale spatio-temporal contexts like remote sensing time series. Bidirectional RNNs (BiRNNs), including BiLSTM and BiGRU variants, have also been explored in imputation and forecasting tasks. By learning from both past and future contexts, these models are particularly useful in cases where the missing values are flanked by available data, as shown in the work conducted by Cao et al. [45]. However, their reliance on full-context availability around missing points makes them less ideal for satellite-based time series, where extended blocks of missing observations are common due to persistent cloud cover.

Recent studies have already combined different gap-filling techniques, such as, Li et al. [46] developed a daily, 0.05° gap-free NDVI dataset spanning 1981–2023 in China by combining valid data identification with spatiotemporal sequence gap-filling techniques. Similarly, Suprijanto et al. [47] applied a new hybrid filter combining multiple reconstruction techniques to recover missing NDVI time series in cloud-prone areas. Our study also follows a similar approach.

The advantage of the Kalman filter (KF) is that, unlike classical filtering methods such as Gaussian smoothing or Savitzky–Golay or Whittaker smoothing, it does not simply perform smoothing based on a fixed window, but explicitly models the process and measurement noise, thus preserving the statistical structure of the time series. While classical smoothing methods are unable to handle uneven time intervals or longer missing data sections due to cloud cover, KF is able to fill in continuous data gaps due to cloud while maintaining temporal coherence. Previous research [31,32] has also shown that KF generally produces lower RMSE/MAE values in the estimation of vegetation indices, especially in cloud-prone areas, than Gaussian or Whittaker smoothing does.

The choice of LSTM network is justified because, although other machine learning methods, such as Random Forest or Convolutional Neural Networks (CNNs), can be applied, LSTM was specifically developed for serial data and is able to capture long-term temporal relationships, which is crucial for modeling vegetation dynamics. Comparative studies [48,49,50] have shown that LSTM-based reconstructions outperform Random Forest and shallow CNN models when the goal is to simultaneously exploit temporal and spatial patterns in high-density time series.

Considering the reasons above, our proposed hybrid approach counterbalances the flexible pattern learning of LSTM with the covariance matrix of KF, thus exploiting the advantages of both worlds. Although the hybrid KF + LSTM method demonstrates the framework on a single index (NDI45), in principle, any physically calibrated multi-channel forecast can be embedded in the KF observation vector.

The full imputation methodology is based on two main assumptions.

(1) The temporal evolution of vegetation intensity can be treated in terms of a five-day lagged period as a time series. The changes in vegetation intensity expressed by the NDI45 value in time t depends on its previous states. Since the Sentinel-2A/B satellite pair returns to the same location every 5 days; this frequency alone is dense enough to model plant physiological changes as a continuous time series. According to a study analyzing the full information content of the vegetation index, the 5-day satellite return for the Sentinel-2 time series from 2017 to 2023 is sufficient to track vegetation dynamics and shows strong temporal dependence between successive observations [51]. Another study investigating rice development stages compared six different lag windows (5–30 days) and showed that the 5-day lag gave the highest (≈88%) time series consistency, while the consistency gradually deteriorated for longer lags [52]. A spatio-temporal image-fusion solution that complements cloud gaps in Sentinel-2 time series has shown that the key to successful reconstruction is the use of immediate temporal neighbor images, because they carry the greatest correlated information about the missing date [53].
(2) In a relatively small area—in our case 20 km × 20 km—the climatic factors are stable as the main dynamics of the NDI45 index is similar to the neighboring locations. Bonthoux and his colleagues [54] found that NDVI time series are positively autocorrelated from 0 to 20 km in 4 km squares in France; within 20 km, Moran-I < 0.2 was found, indicating sufficient statistical correlation between adjacent sites. In the Central Highlands watershed of Taiwan, Chu and his colleagues [55] showed that NDVI is strongly correlated (Moran-I > 0.3) up to 2.8 km, and the correlation decays gradually rather than abruptly, suggesting that relatively topographically homogeneous areas evolve under common climatic driving forces.

Of course, neither of the assumptions is valid without restrictions. Extraordinary events, such as forest or grassland fires, can cause significant, discrete jumps in the index within a short time, and the phenological curves of intensively irrigated agricultural plots can also deviate from their natural vegetation. Aware of all such possibilities, we treated the two premises as first-order, complementary working hypotheses, which represent the starting point of the model-based analysis. We used correlation-oriented modeling to map local differences and zone-specific dynamics; the methodological details of this are described in Section 2.3.2.

#### 2.3.1. Modeling Local Temporal Dependencies with the Kalman Filter

For modeling the temporal dependencies inside the time series, we used the Kalman filter which proved to be a versatile method for gap imputations [56]. The advantage of the method is that it simultaneously takes into account the effects of the system dynamics model (process noise) and measurement noise within the stochastic state–space model, so that when estimating missing values, it not only performs a simple interpolation between observations, but also tries to preserve the statistical structure of the series as a whole [57].

When applying the Kalman filter, the missing blocks are filled using iterative Expectation–Maximization steps: first, a prediction is generated based on previous estimates and the reference series, and then the estimate is refined by combining the incoming observations and their uncertainty (“update”). This two-phase cycle—prediction and correction—allows the filter to handle both short- and long-term trends and seasonal relationships, thereby minimizing temporal distortions when filling in the gaps [58]. The Kalman filter is particularly advantageous when the gaps in the series do not occur at a single point, but in connected blocks (“gaps”), since the filter is inherently able to “fill in” entire blocks in a way that maintains data consistency. Furthermore, it is known from comparative studies that the Kalman filter produces lower errors (RMSE, MAE) compared to traditional, simpler methods (e.g., linear interpolation, KNN) [59].

Kalman filter methods developed for the gap filling of vegetation indices use an explicit linear state transition, where the “a priori” estimate of NDVI/NDI45 is a linear function of the previous time state [31].

In this research, we defined a state–space model which was passed to the Kalman filter in order to make the imputations. The model described formally by Equations (2)–(5) considers three elements that can be present in a time series: the trend, the seasonality, and observation error. In our case, each of these elements could be important: local and global trends could appear due to growing seasons or strengthening aridity, annual seasonality is regularly present, and noisy observations also occur.

(2) $${NDI45}_{t}=L_{t}+S_{t}+\eta_{t,4},\;\eta_{t,4}\sim N(0,\theta_{4})$$

(3) $$L_{t+1}=L_{t}+\beta_{t}+\eta_{t,1},\;\eta_{t,1}\sim N(0,\theta_{1})$$

(4) $$\beta_{t+1}=\beta_{t}+\eta_{t,2},\;\eta_{t,2}\sim N(0,\theta_{2})$$

(5) $$S_{t+1}=-\sum_{j=1}^{s-1}S_{t+1-j}+\eta_{t,3},\;\eta_{t,3}\sim N(0,\theta_{3})$$

where,

${NDI45}_{t}$—NDI45 value at time t;
$L_{t}$—level (trend) component value at time t;
$S_{t}\;$—seasonal component value at time t;
$\beta_{t}$—slope component value at time t;
$\eta_{t,x}$—Gaussian disturbance term associated with component x;
$\theta_{1}$—variance of the level disturbance; controls how wiggly the long-term mean of NDI45 can be;
$\theta_{2}$—variance of the slope disturbance; governs how quickly growth/decline trends may accelerate;
$\theta_{3}$—common variance for all seasonal disturbances; determines how tightly the seasonal cycle adheres to its perfect sinusoid;
$\theta_{4}$—observation error variance; reflects measurement noise and any sub-pixel variability not captured by the state.

To determine the noise level parameters of the space–state model defined for the Kalman filter, we used the existing scientific and technical literature. There are limited studies and scientific research available for the definition of the NDI45 index; therefore, where we did not find literature on this, we worked based on the reasoning applied to the NDVI index. For the process-noise variance of the level component, we adopted the empirical relationship σ = 0.07⋅RMS (NDI45) reported by Forkel et al. [60] for 15-day composites in the Pampas region, scaled to the 5-day temporal resolution of our Sentinel-2 series. The slope disturbance variance was set to one-fifth of the scaled level variance to reflect the derivative relationship between level and slope. The variance of the seasonal component was fixed experimentally at 0.0011, which maintained 95% confidence interval coverage for both complete and incomplete series.

To estimate the observation error, the ±1.5% relative radiometric accuracy (ε = 0.015) of the Sentinel-2 L2A product was propagated to the NDI45 index using the relation expressed by Equation (6). We obtained a 0.0104 value, which was introduced into the model as a constant measurement standard deviation.

(6) $$\sigma =\frac{\varepsilon }{\sqrt{2}}(1-\bar{{NDI45}^{2}})$$

#### 2.3.2. Modeling Spatial Similarities of NDI45 Dynamics with LSTM

Vegetation indices are determined by climate, phenology, land use, and management practices, which interact in a nonlinear manner and can vary over months or even years. LSTM’s (long short-term memory) “memory cells” are versatile in learning these kinds of relationships [48,49,50].

**Figure 3:**
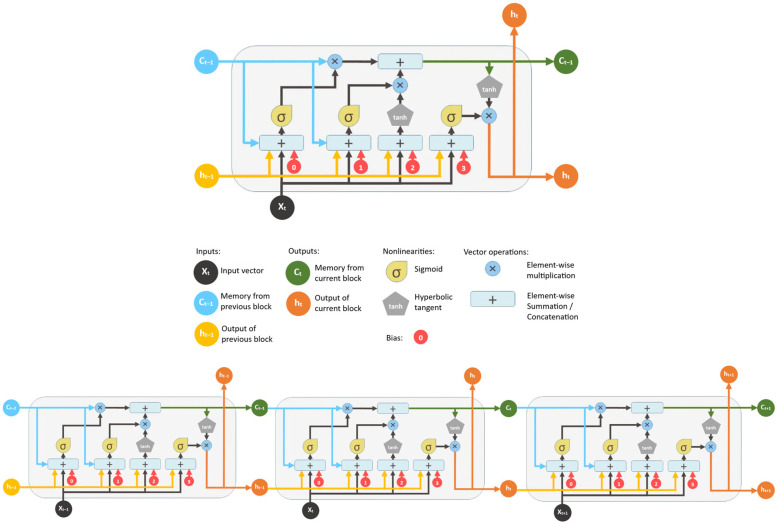
The architecture of an LSTM cell and an LSTM network (source: https://blog.mlreview.com/).

LSTM (long short-term memory) is a special recurrent neural network (RNN) layer specifically designed to learn long-term dependencies. Each LSTM cell contains three main gates: the forget gate decides how much of the previous cell state is retained; the input gate controls how much new information is allowed from the current input; and the output gate determines how much of the new cell state is output to the next recursive state. Each of these gates uses a sigmoid activation function to weight the inputs and the previous hidden state, thus adaptively controlling the flow of information (Figure 3).

The cell state itself is the “memory” vector, which is updated by linear and nonlinear operations (element-wise multiplication with the “gates”, addition with new candidate values, tanh to generate new values). This structure allows the LSTM to simultaneously learn to preserve long-term trends—with limited information output from the output gate—and quickly adapt to short-term changes using the input gate. The resulting memory cells are particularly effective for modeling complex, nonlinear, temporal dependencies, as is often the case with vegetation indices.

Although our study area is relatively small, due to the reduced length of the time series, no single, general LSTM model is able to map equally well different types of area coverage (e.g., vegetation, built-up areas), and even the learning of differences within vegetation. Therefore, we performed a correlation-based clustering in advance: cells with similar temporal dynamics were classified into a cluster. This was performed with a weighted Pearson correlation coefficient whose weights equal the number of jointly available, non-missing data points. Thus, greater emphasis was given to those cells that provided a longer, and thus more representative, common section for the correlation analysis (Equation (7)).

(7) $$r_{wi}=cor(x_{r},x_{i})\cdot \frac{L_{i}}{L_{r}}$$

where,

$r_{wi}$—weighted correlation for cell i;
$x_{r}$—time series at the reference location (longest series without gaps);
$x_{i}$—time series at location i;
$L_{r}$—number of valid data in the time series at the reference location;
$L_{i}$—number of valid data in the time series at location i.

The clustering algorithm iteratively performed the following two steps until all the studied sites were assigned to a class:

− it selects the site that does not yet belong to any class and that has the least amount of missing data.
− it finds the sites with which the weighted Pearson correlation value exceeds a predefined threshold (in our case 0.75) and which are also not yet part of any class, and then forms a new class from them.

During time-series LSTM embedding, it was crucial to preserve the temporal order of the data: the data must not be shuffled randomly, as the network learns the temporal relationships between the samples. If we swap the chronological structure during the train–validation phase, the model will not be able to track the dynamics of the series, and the learning will be meaningless.

After the learning phase for each cluster, the prediction was performed using a hybrid interpolation strategy. This strategy combines two sources of information:

− observed values—if the original NDI45 value is available at the given time step (not NaN), then this actual measurement is scaled and used directly as input to the model.
− model predictions—if the original value is missing, but a prediction was already made in a previous iteration for the same time point, then this scaled prediction is incorporated.

This step-by-step approach ensures that the model always receives the most reliable information: first we prioritize real measurements, then we use our own predictions where necessary, and only finally rely on the simplest interpolation. This way, the network makes maximum use of the actual data and only “fills in” missing points where data is truly unavailable, while working with a complete input series at every time step.

#### 2.3.3. The Ensemble Model

The two imputation methods presented above—the LSTM-based approach and the Kalman filter gap filling—complement each other with different philosophies in filling in the missing data. We created the final imputed dataset by using the outputs of these two methods together. Our basic goal was to have the LSTM model capture the general patterns of vegetation dynamics as completely as possible—those characteristics that are most likely to exist in other cells that are highly correlated with the cell with the most complete dataset—while the Kalman filter serves for the local characteristics of individual cells and fine corrections.

To combine the two imputations, we used the following weighting principle: for each cell at each time moment, we assigned two uncertainty measures [61]: the value of the Kalman filter forecast covariance element (P_k_) taken from the diagonal of the posterior covariance matrix and the standard deviation of the LSTM model forecast error (σ_LSTM_), computed from the difference between LSTM predictions and available cloud-free observations during model validation. Both quantities are expressed in NDI45 units, enabling direct comparison. Based on these, the final, weighted value is obtained, $\hat{{NDI45}_{t}}$, where the weights (w_KF_, w_LSTM_) are given so that smaller uncertainties are given greater weight (Equations (8) and (9)).

(8) wLSTM=1σLSTM21σLSTM2+1PkwKF=1Pk1σLSTM2+1Pk

(9) $$\hat{NDI45}=w_{KF}{NDI45}_{KF}+w_{LSTM}{NDI45}_{LSTM}$$

Thus, the LSTM estimate dominates if its forecast has a small variance, while the Kalman filter correction prevails if the Kalman squared uncertainty for a given cell is lower. This fusion procedure ensures that the final result simultaneously preserves global and local corrections.

If the KF and LSTM estimates have (approximately) independent or weakly correlated errors and their variances are known, then precision-based weighting yields the minimum-variance (best) linear unbiased combination (BLUE). The Kalman update itself is inherently a precision-weighted average, which means that using P_k_ from the KF and σ^2^_LSTM_ from the LSTM as weights is both natural and theoretically well-founded for fusing the two estimates [62,63].

All the simulation steps of the study—from test filtering on the time series of the seven representative sites, to Kalman filter gap filling covering the entire South American Pampas, and to the spatial calculation of the reliability and coverage uncertainty metrics—were implemented entirely with in-house-developed software running in the R 4.5.1. environment. During the project, we not only relied on standard packages (such as dlm, terra, netcdf4), but also created several R functions and classes written specifically for this task: such as the adaptive noise parameter generator, the KF–LSTM weighting module, the parallelized, tiled gap-filling library, and the reliability mapping routine based on posterior covariance. We implemented the training of the LSTM networks and the predictions based on them in Python 3.11, using the TensorFlow framework, and then integrated the obtained results into R for the KF-based fusion. These components allow us to handle datasets consisting of hundreds of time steps and millions of pixels in an efficient manner, and to automate the entire processing chain—from data preparation to statistical validation. In other words, purpose-specific software development was essential for the analysis: the synergy of the R and Python-based infrastructure and our own algorithms ensured that the theoretical framework of the methodology became a working, large-scale tool. In some cases, we also used CDO (Climate Data Operator) commands to merge layers, e.g., to create the final weighted model.

#### 2.3.4. Evaluation Metrics

The performance of both the Kalman filter and LSTM models was evaluated quantitatively by comparing reconstructed NDI45 values against actual cloud-free observations in the time series. For each pixel, only those dates with valid Sentinel-2 measurements were used to compute the evaluation metrics, ensuring a fair and consistent basis for comparison between models. This procedure was applied separately to the outputs of the Kalman filter and the LSTM, enabling a direct quantitative assessment of each method’s reconstruction capability.

Four indicators were used to spatially characterize precision and the uncertainty of the reconstruction: mean average error (MAE), mean absolute percentage error (MAPE), root mean square error (RMSE), and reliability. The aim was to simultaneously measure the precision of the reconstruction and the uncertainty with which the model provides values at the pixel level.

Methodological studies confirm that MAE is Bayes-optimal [64,65] and the use of it whenever the residuals are non-normal or heavy-tailed, which is typical for environmental time series with outliers and skew. Gap-filling studies echo this practice. Hird and McDermid reconstructed noisy NDVI curves, and they ranked six smoothing filters solely with MAE and the Pearson correlation between reconstructed and reference series [66]. Kandasamy et al. compared eight gap-filling algorithms for MODIS leaf-area-index time series and again relied on the twin criteria of low MAE and high Pearson *r* to decide which method worked best [67]. Its value was calculated for each pixel and each such time step, and then the values were averaged (Equation (10)):

(10) $$MAE=\frac{1}{T}\sum_{t=1}^{T}\left|y_{t}-x_{t}\right|$$

where,

y_t_—actual observation;
x_t_—reconstructed value;
T—number of time steps.

MAE directly measures the average magnitude of reconstruction error without being overly influenced by occasional large deviations, making it well suited for environmental time series that may include outliers or heavy-tailed error distributions. In the NDI45 gap-filling context, a low MAE indicates that the reconstruction closely matches actual cloud-free observations, which is critical for preserving the sensitivity of NDI45 to the vegetation conditions, particularly in high-LAI crops where small differences can signal real yield changes.

MAPE gives the relative error in percentage form. This metric expresses the magnitude of the errors as a proportion of the measured values, making it easy for users to interpret [68]. MAPE is particularly suitable for applications where the relative deviation of the estimate is important, such as in the assessment of the gap filling of vegetation indices [69,70].

$$MAPE=\frac{100}{T}\sum_{t=1}^{T}\left|\frac{y_{t}-x_{t}}{y_{t}}\right|$$

RMSE is the square root of the mean square of the differences between the predicted and observed values. This metric is sensitive to large deviations, thus indicating the presence of significant errors [71]. In the case of spatial and temporal interpolation of vegetation indices (e.g., NDVI, EVI), RMSE is a frequently used reference for comparing different models [72,73].

$$RMSE=\sqrt{\frac{1}{T}\sum_{t=1}^{T}{\left(y_{t}-x_{t}\right)}^{2}}$$

The simultaneous use of the above three indicators allows for the simultaneous evaluation of the absolute magnitude (MAE, RMSE) and relative significance (MAPE) of the errors, thus providing a comprehensive picture of the performance of the interpolation method in reconstructing vegetation indices.

The reliability is the time series average of the diagonal elements of the posterior covariance matrix ($\sigma_{t}^{2}$) of the Kalman filter. For a given pixel, it can be expressed as presented in Equation (11)

(11) $$R=\frac{1}{T}\sum_{t=1}^{T}\sigma_{t}^{2}$$

MAE directly shows how much the reconstruction deviates from the true NDI45; reliability shows how sure the model is of its own estimate. Together, these two indicators can separate the areas of high accuracy–low uncertainty and low accuracy–high uncertainty, which is important for decision support purposes.

## 3. Results

### 3.1. Modeling Local Temporal Dependencies with the Kalman Filter

In the first step of this study, during the imputation of incomplete time series based on the Kalman filter, the problem of removing extreme values from the series arose. Since every real observation, even an extreme one, carries real information about the true state, discarding it forces the Kalman filter to base its update on an incorrect probability and introduces systematic bias. Extreme observations often indicate that the hidden state has jumped or drifted more than usual (e.g., sudden vegetation growth after heavy rain). By deleting these data points, the filter pulls the posterior mean back towards its prior prediction, thus never “learning” the true magnitude of the change. In addition, the covariance update of the filter depends on the size of the residual. Large residuals legitimately increase uncertainty, which forces the filter to widen its confidence bands. Removing extreme values results in overly reliable error bars that can mislead the further weighting process.

The process level was set based on the Pampas study by Forkel and his colleagues [60] who found an empirical relationship of σ = 0.07 · RMS (NDI45) for 15-day composites. Since our Sentinel-2 time series have a 5-day resolution, the 5-day standard deviation was divided by $\sqrt{3}$ due to the time step scaling. The trend slope is a daily derivative, so the standard deviation of the slope noise was reduced to one-fifth of the 5-day level value. The noise of the seasonal component was experimentally fixed at 0.0011 (Table 1); this proved to be the value that maintained the 95% confidence interval coverage even for the longest, complete time series, while also resulting in minimal uncertainty for shorter or incomplete series.

**Figure 4:**
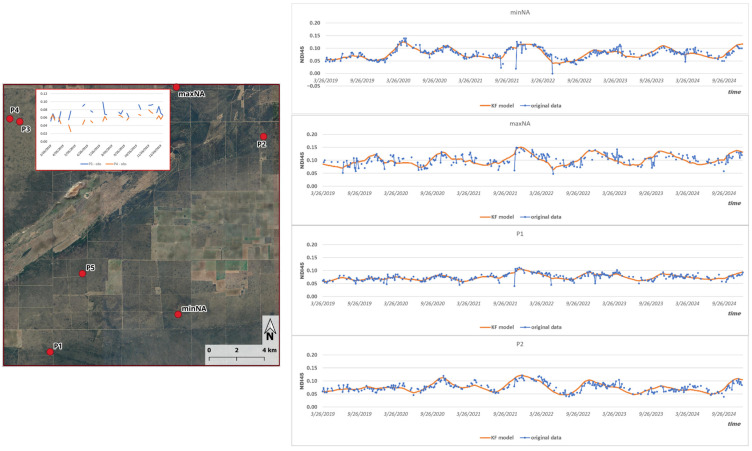
Location of the seven test sites inside the study area along with Kalman filter imputation model over the observed data.

The developed parameterization was tested at seven test sites: the most complete (minNA) and most missing pixels (maxNA), and five randomly selected sites (P1–P5) as presented in Figure 4.

Although sample sites 3 and 4 are only a short distance apart, geographical proximity alone does not guarantee the correlation of the NDI45 time series. The cloud-induced data gaps coincide in time, but the index dynamics for each day may even show opposite phases at the two locations, as is the case for 2019.

**Figure 5:**
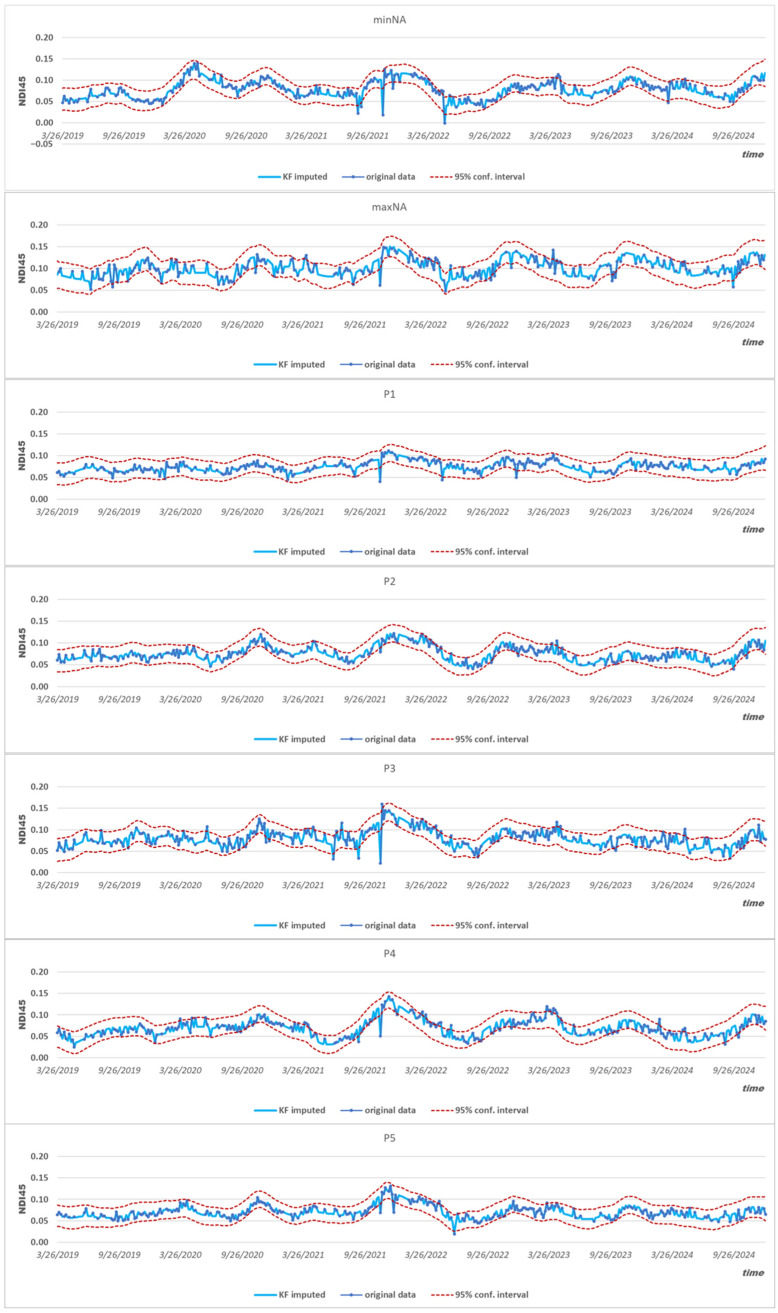
Combined (gap-filled) time series based on KF imputation.

The normally distributed 95% confidence intervals calculated from the posterior variances from the Kalman filter closely follow the real observations. In each case, 95% of the recorded values lie within the estimated band. The complete set of time series imputed for the missing time points and concatenated from the actual measured data (Figure 5) served as the basis for generating the spatial fields of the NDI45 index for the research area.

**Figure 6:**
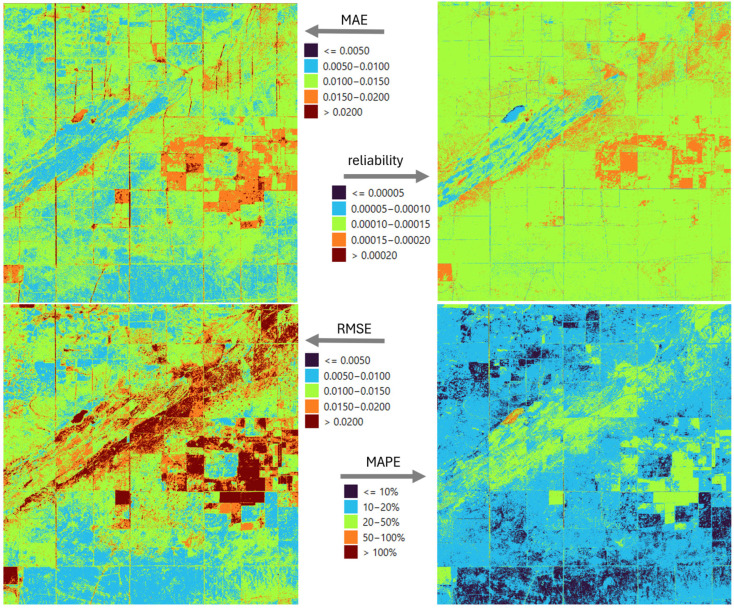
Error metrics (MAE, MAPE, RMSE) and reliability spatial distribution in case of KF model.

Four indicators were used to spatially characterize precision and the uncertainty of the reconstruction: MAE, RMSE, MAPE, and reliability (Figure 6). The mean absolute error creates clearly distinguishable patchy patterns in the study area. Although the deviations are orders of magnitude lower than the values of the index itself, they also highlight the limitations of the Kalman filter’s spatial state model. RMSE shows a similar error pattern to MAE, but it can be observed that stronger errors appear on the main diagonal of the square of the study area and in its northeastern part. The explanation for this is that extreme values occurring in seasonal dynamics are more difficult for the filling model to predict, so rare but significant errors occur. Thus, an erroneously high filled value causes an outsized difference in the proportions. A similar effect occurs in pixels with heterogeneous surface cover, where the spectral mixture changes rapidly in time, which drastically increases the filling error at certain times. The above comments are also consistent with the spatial results shown by reliability, where high RMSE values also have greater uncertainty. The dynamic characteristics of the vegetation, and in particular the different timing of the vegetation periods by crop, heterogeneously affect the applicability and adaptive performance of the unified model.

In the studied area, marked spatial variations of this indicator can also be observed, which differ in their pattern from the spatial distribution represented by average error.

**Figure 7:**
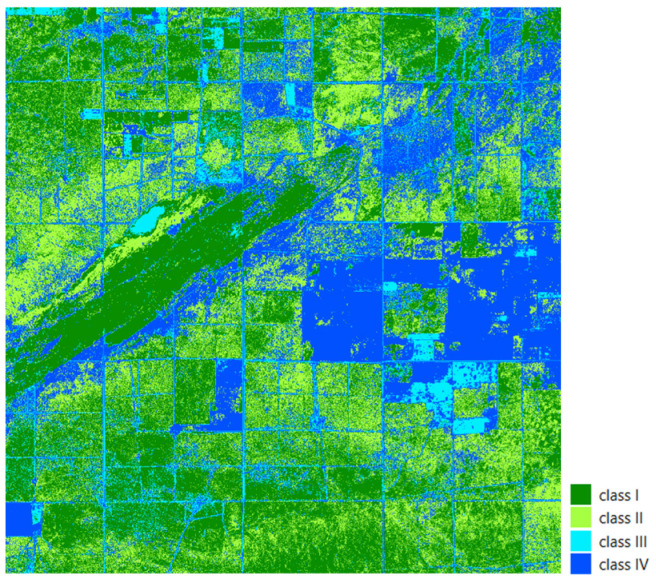
Classes obtained after cross tabulating threshold split mean absolute error and mean variance.

We defined four categories by cross tabulating the mean absolute error and reliability values in the study area (Figure 7). The absolute error is considered low if its value is less than 0.012 and high if it exceeds this threshold; reliability is low if it is below 0.000125 and high if it is above it. The first group (class I) includes pixels with both low mean error and low variance: in this range, the Kalman filter effectively models vegetation dynamics, and the process and measurement noise are low. The second category (class II) is a combination of low average error and high variance: here, sufficient data are available to correctly determine the average value, but uncertainties arising during the modeling—such as variable vegetation patterns or local environmental effects—result in a wide confidence band, so although there is no systematic deviation, the accuracy of the estimate is variable. The third case (class III) is characterized by a high average error but low variance: this is a typical case of systematic bias, when the Kalman filter consistently estimates in a different direction due to environmental covariates missing from the model—such as soil type, topography, or plot boundaries—but “confidently” sticks to its own, incorrect state model.

Finally, the fourth category (class IV) includes high mean error and high variance: here, due to spatial heterogeneity, the intersection of data mosaics, or the sparse distribution of sampling points, the model neither fits exactly nor maintains a consistent estimate, so over- and under-estimations are common.

**Figure 8:**
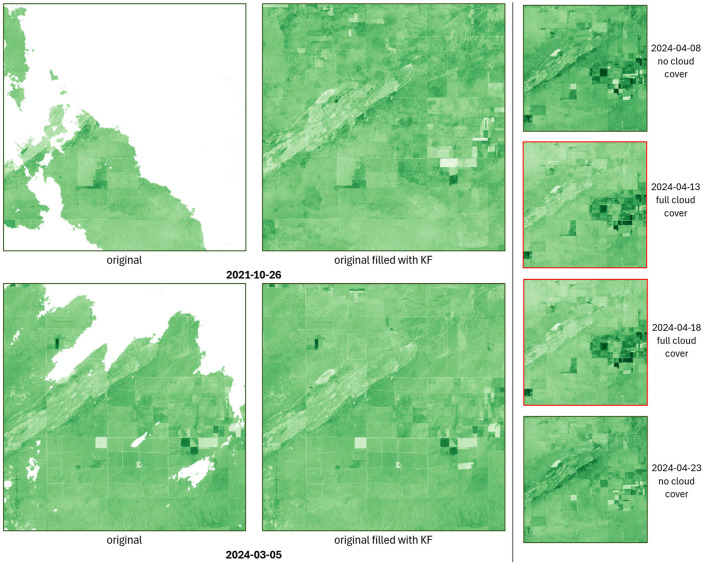
Gap filling results for partial data missingness (**left**) and total data missingness (**right**).

The dataset produced by the gap-filling procedure applied to replace missing data effectively reconstructs the missing spatial values of the NDI45 index and provides reliable estimates of the index values even for full days in the case of full cloud cover (Figure 8).

### 3.2. Modeling Spatial Similarities of NDI45 Dynamics with LSTM

Our second model for the gap-filling task was a long short-term memory (LSTM)-based network. To train the models tailored to each time series, corresponding to each location, we first had to quantify the similarity between the time series.

**Figure 9:**
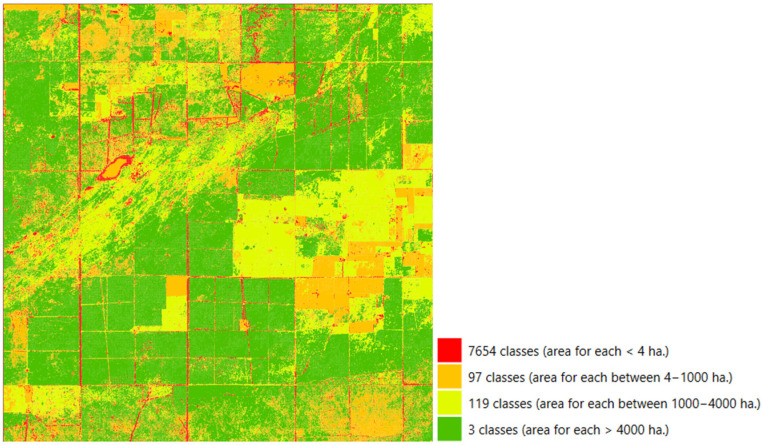
Clustering based on weighted correlation of time series.

Based on the spatial distribution of the resulting classes (Figure 9), we observed that the three largest clusters cover more than 54% of the study area. This indicates that important similarities exist in the NDI45 index, and this vegetation indicator is able to correlate over extensive areas. It is often observed that a high degree of time series similarity is also coupled with spatial proximity; however, there are also many cases where similar time series appear at sites that are significantly distant from each other. In our view, therefore, for spatial autocorrelation, it is more useful to use the approach that we have applied, which is based on similarity as demonstrated by time series correlation. Only 0.4% of the locations (97.2% of the total number of clusters) are represented by clusters with a size of less than 100 points, suggesting that the weighted correlation threshold of 0.75 is too strict for them. These areas most often occur along roads, where the presence of vegetation is not constant.

For the resulting classes, we trained the LSTM model on the cell with the least missing data in the group. The list of used parameters for LSTM is presented in Table 2.

In the case of long short-term memory (LSTM) networks, typically several thousand training samples are available, while in our case we processed only 422 time-step series, which makes the model particularly susceptible to overfitting. We addressed the problem by early stopping: during training, we continuously monitor the validation loss, and as soon as it reaches a minimum, i.e., the model’s generalization ability is at its best, we interrupt training. This prevents the network from “cramming” on noise or irrelevant features of the training set, which could degrade its performance on new, unknown data.

**Figure 10:**
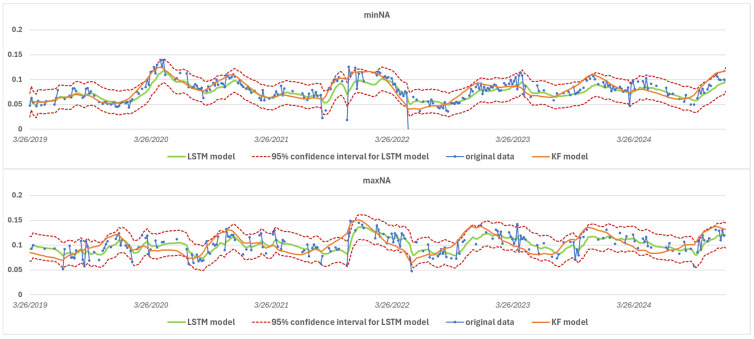
Interleaving of LSTM and KF model imputation for the most and least complete time series location.

Although Figure 10 shows data collected at two different geographical locations, the pattern of the curves obtained at both points is similar, suggesting spatial homogeneity of the hydro-meteorological and vegetation cycles in the study area. This similarity between the sites, i.e., a certain degree of uniformity in the causal factors, justifies the correlation analysis previously carried out between the data series and supports the need for data imputation.

**Figure 11:**
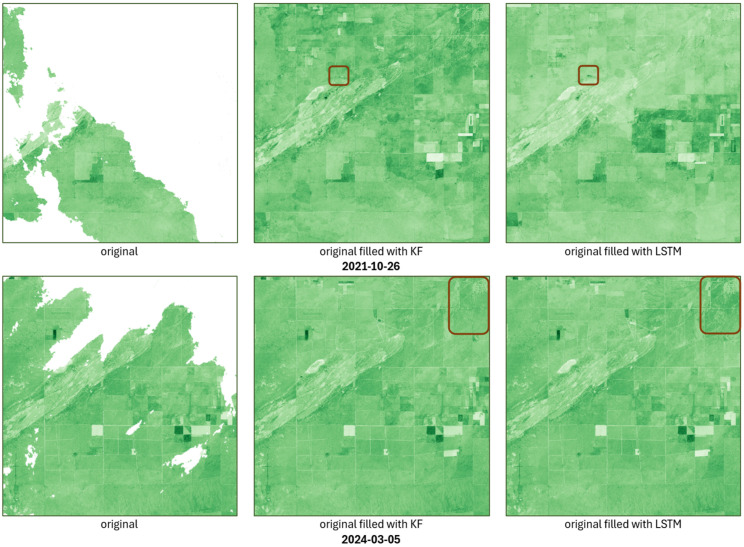
Spatial result of KF and LSTM models, highlighting the differences between them (outlined in brown rectangles).

The spatial results also clearly reflect the differences between the two approaches. For both 16 October 2021 and 5 March 2024, the LSTM model predicts lower NDI45 values at most of the locations affected by incomplete data, but at some points, the opposite is observed, as is well illustrated by the highlighted areas in Figure 11.

**Figure 12:**
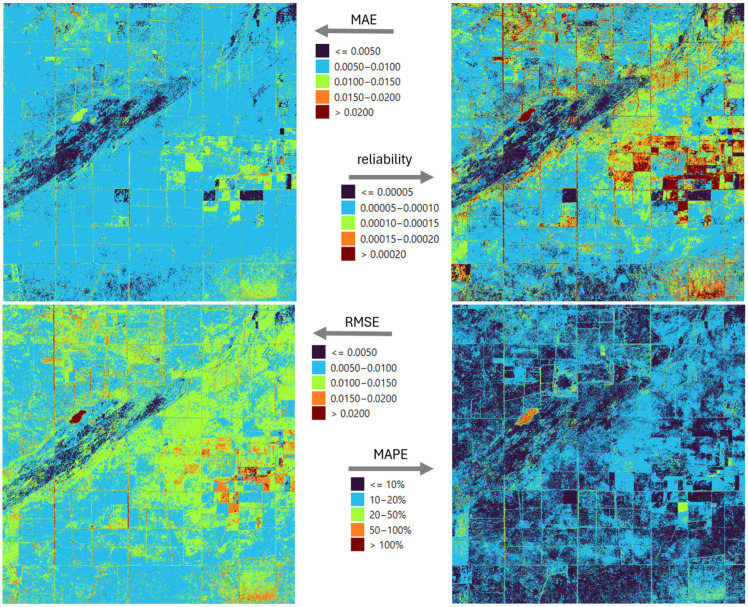
Error metrics (MAE, MAPE, RMSE) and reliability spatial distribution in case of LSTM model.

A detailed analysis of the errors of the LSTM model shows that this non-deterministic approach is in most cases a more precise approximation of the true values than the Kalman filter (Figure 6 vs. Figure 12). However, it is clearly observed that the structure of the error patterns remains unchanged, which may be supported by the temporal variation in land use and the specific dynamics of the vegetation. Notably, MAPE values are below 20% at almost all locations, and below 10% at 47% of locations. Since MAPE is highly sensitive to values close to zero, this explains the exceptionally high error percentages in Figure 6 and Figure 12 for the lake in the western part of the study area, which appears as an orange-hued spot in these figures.

**Figure 13:**
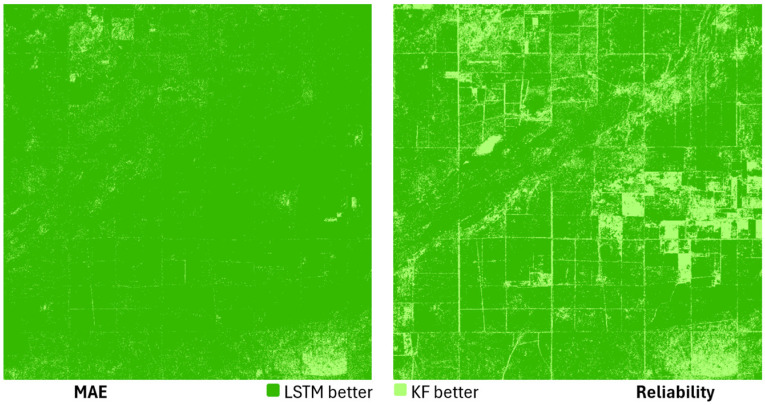
Comparison of KF and LSTM model performance based on MAE and reliability.

In the study area, LSTM alone achieved lower MAE values than KF at most locations, reflecting its ability to capture nonlinear temporal patterns, while KF generally produced more spatially homogeneous reliability maps with lower variance in its uncertainty estimates (Figure 13). On average, KF MAE values ranged from 0.012 to 0.018, while LSTM MAE values ranged from 0.010 to 0.016. The ensemble model combined these strengths: MAE values were further reduced to the range of 0.009–0.014, and reliability improved in many areas compared to using LSTM alone. This confirms that the variance-weighted mixing approach effectively exploits the complementary advantages of both models, resulting in higher accuracy while maintaining or improving the reliability of the predictions.

### 3.3. Modeling NDI45 with the Ensemble Model

**Figure 14:**
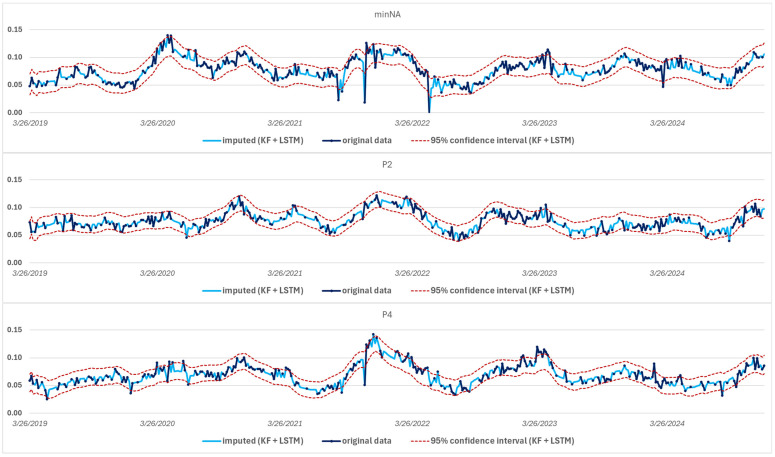
Ensemble model results combined with original data.

The results from above have shown that both the Kalman filter and the LSTM model contribute specific strengths to fill in the missing data. Therefore, we combined the results of the two approaches by using the variance of the errors produced by the models as a weighting factor. The weights were calculated as given in Equations (7) and (8), so that the model with lower variance (i.e., less uncertainty) contributes more to the final estimate (Figure 14).

When the two models are merged, the combined confidence intervals are determined using Equation (12) in which w_KF_ and w_LSTM_ are the weights of the models, σ_KF_ and σ_LSTM_ are the standard deviation of errors, and Cov(e_KF_, e_LSTM_) is the covariance of the errors.

(12) $$\sigma^{2}=w_{KF}^{2}\sigma_{KF}^{2}+w_{LSTM}^{2}\sigma_{LSTM}^{2}+2w_{KF}w_{LSTM}Cov(e_{KF},e_{LSTM})$$

**Figure 15:**
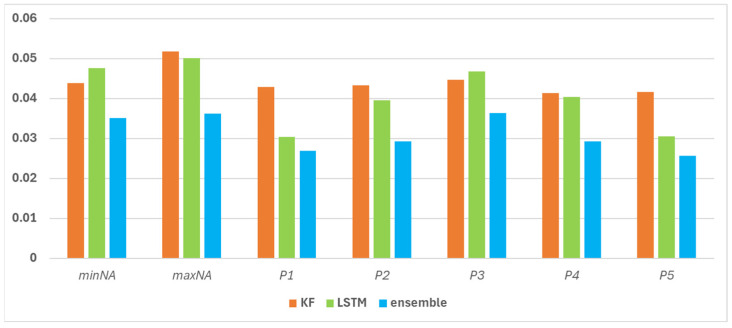
Average width of confidence intervals for the KF, LSTM, and ensemble models.

An examination of the average confidence intervals for different locations in the study area clearly indicates that the combination of the two models significantly improves the estimation accuracy (Figure 15). While both methods alone produce reliable results, when combined, they complement each other in a complementary way, according to the pre-defined variance weighting, and thus contribute to a more accurate imputation of missing values with narrower, tighter confidence intervals.

**Figure 16:**
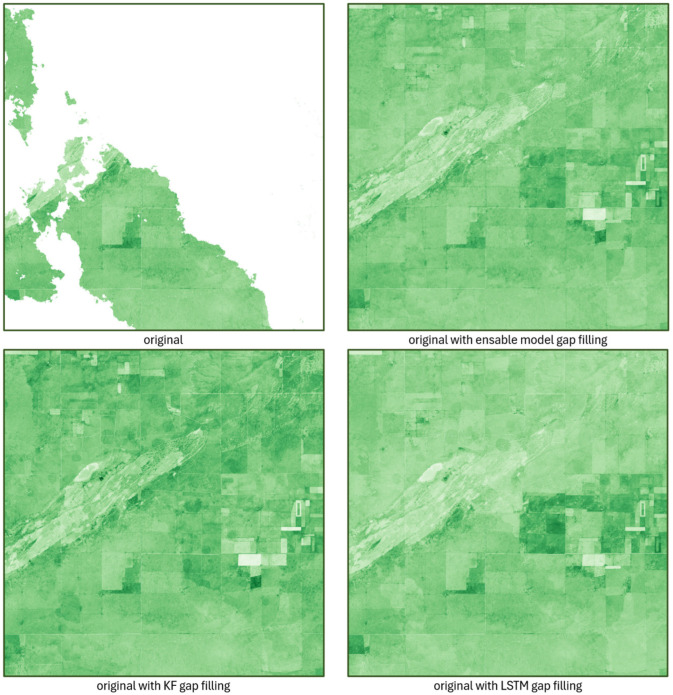
Imputation results for KF, LSTM, and ensemble models for image taken on 16 October 2021.

From Figure 16, it can be observed that both the Kalman filter (KF) and the LSTM model alone can effectively fill in the missing areas; however, there are differences in shading (value) along the imputed values, especially at the boundary of the existing and imputed regions. For example, if a cloud obscures half of an agricultural parcel, a sharp break often appears along the edge of the data gap along the independent imputation of the KF or LSTM. In contrast, the proposed ensemble model smooths these boundaries, yielding more uniform NDI45 values while preserving spatial homogeneity.

**Figure 17:**
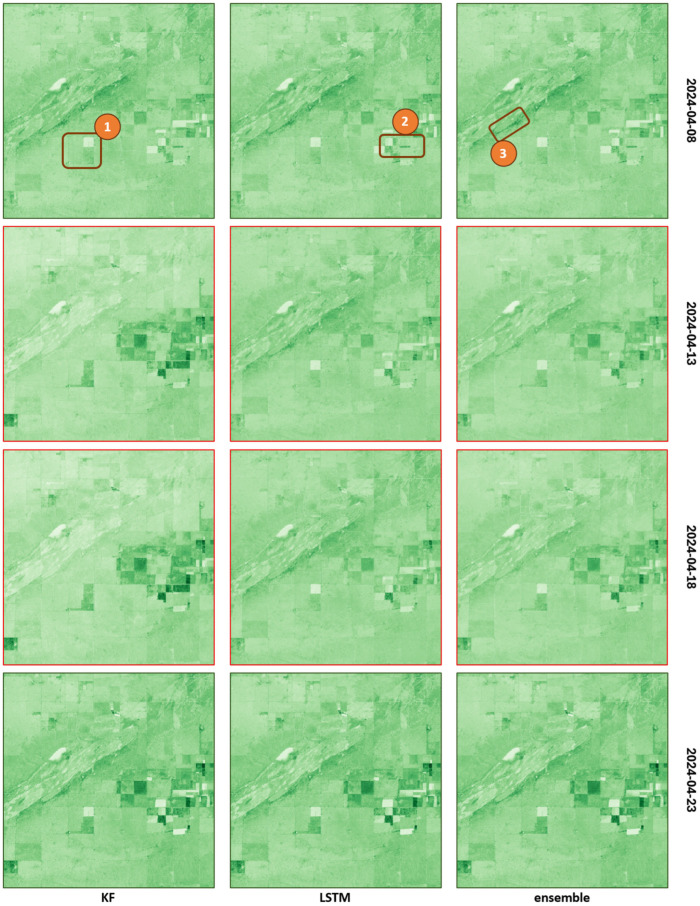
Days with completely missing data imputed by KF, LSTM, and ensemble models (numbered brown rectangles indicate the discussed sites).

In the case of a complete lack of data at a given moment in time, the two models can compensate for each other’s weaknesses. The first and last rows of Figure 17 each show the actual images of identical cloud-free reference dates, while the middle two rows show the imputed images generated by the KF, LSTM, and ensemble models. It can be clearly observed that at sites 1 and 2, the KF model strongly overfits (especially at site 2, where the imputed NDI45 values are even higher than the values of the cloud-free initial and final states), while at site 3, the LSTM model shows overfitting. In both cases, however, the imputation of the other model contributes to the final ensemble model estimates being closer to reality.

**Figure 18:**
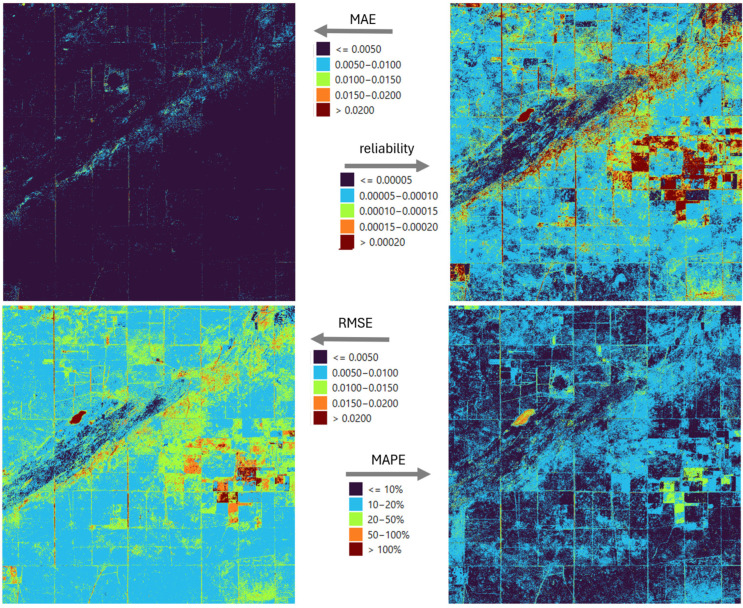
Error metrics (MAE, MAPE, RMSE) and reliability spatial distribution in case of the ensemble model.

The ensemble approach is able to reduce the errors of both the KF and the LSTM (Figure 18). Examination of the MAE values shows that the majority of the total study area fell into the lowest error category, i.e., the combined model was able to refine the KF results, which were previously characterized by higher error, compared to the LSTM (Figure 6 and Figure 12). In the case of the ensemble model, the RMSE and MAPE show similar behavior compared to the LSTM results. On the one hand, a decrease in error values can be observed in most of the study area, but in the eastern part, in about two plots, a slight increase can also be observed. In terms of reliability, the LSTM alone has already outperformed the KF at most points, but the ensemble model allows for further improvements in this area as well, with higher reliability at many locations, although there are also areas where the degree of error reduction is accompanied by an increase in confidence, i.e., although the accuracy of imputation has improved, the uncertainty of the model has increased in relative terms.

**Figure 19:**
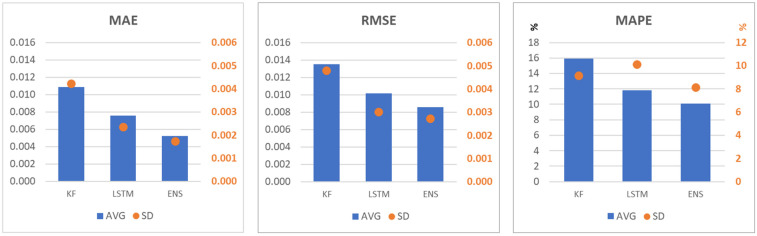
Spatially averaged error metrics (avg) with their standard deviation (sd) in case of the three gap filling models.

In the case of the error metrics averaged over the study area, these have lower values in the ensemble model than in the case of the KF or LSTM separately (Figure 19).

## 4. Discussion

Running the Kalman filter and LSTM together cut the mean absolute error by about 25–35% and tightened the 95% confidence band by roughly 25–40% compared with using each model on its own. In high-leaf-area crops, even a small shift in the NDI45 index can signal a real change in yield, so this drop in error matters. Each model has its importance in the gap-filling process. We combine the two by giving more weight to the one that is more certain at each time step. This keeps the Kalman filter’s steadiness and the LSTM’s flexibility.

Compared to these traditional approaches, our variance-weighted KF–LSTM ensemble offers both accuracy and uncertainty estimation advantages. Classic gap-filling tools such as HANTS, IDR, and EGF-WS reach 10–20% error in cloudy tropics but rarely give formal confidence limits and as noted by Julien and Sobrino [24] and Kandasamy et al. [67], they can underperform in heterogeneous landscapes or under rapid phenological transitions. Deep-learning setups like CNN-RNN, GRU-D, or Temporal Fusion Transformers can push the error lower, yet they need very dense training data and often skip pixel-level reliability. Our method deals with both gaps: (1) it builds a seamless 20 m, five-day NDI45 series from a single sensor, and (2) it attaches an uncertainty value to every pixel and date, taken straight from the posterior variance. The inclusion of model-specific uncertainty in the weighting process allows the ensemble to adapt dynamically in space and time, giving greater influence to the more reliable model for each pixel–date combination. This adaptability, combined with the ability to retain sharp changes in vegetation conditions, makes the approach more robust for operational agricultural monitoring in environments with frequent cloud cover and high inter-annual variability.

In the context of the Pampas, where large, high-LAI crops such as soybean, corn, and wheat dominate, a 25–35% reduction in the MAE of the ensemble model and a 25–40% narrowing of the 95% confidence interval have clear operational value. More accurate and reliable NDI45 time series could improve early detection of water shortages and pest outbreaks, allowing for timely interventions. This is particularly important in years with pronounced ENSO-driven rainfall variability, where even short delays in management decisions can result in significant yield losses. The combination of increased temporal continuity and explicit pixel-level uncertainty also supports the calibration of crop growth and yield models, refinement of insurance loss assessments, and targeted use of precision agriculture inputs. By ensuring that spatial patterns of vegetation condition are consistently recorded during cloudy periods, the combined approach strengthens the reliability of long-term monitoring datasets used by farmers and regional agricultural agencies.

The Kalman-only run tracks the season well without big over- or under-shoots. It does smooth out rare, sharp spikes, which is expected for a general filter. When data are missing, the two models swap roles. In Figure 18, the Kalman filter overshoots in sites 1 and 2, while the LSTM overshoots in site 3. The hybrid mix pulls the final value closer to reality in both cases. The curves of the two models cross in a regular rhythm, showing that each takes the lead when it has more confidence.

The weighted Pearson threshold of 0.75 was chosen for practical reasons, not based on formal prior research. Our goal was to obtain clusters that were homogeneous enough for the LSTM to learn, but statistically large enough. We did not perform formal sensitivity testing in this work, as such an analysis was beyond the scope and primary goal of this study, which is to present the hybrid reconstruction method. The threshold value of 0.75 clearly delineates the boundaries of crop fields separated by the road network, and small clusters appear predominantly in these—roadside—areas. This conservative compromise ensures that the pattern learned by the LSTM at one location can be reliably transferred to pixels with similar properties.

The main disadvantage of using a fixed Pearson correlation threshold is that it applies the same similarity criterion to all sites, regardless of the local variability in vegetation dynamics. In homogeneous areas, a high fixed threshold may unnecessarily fragment clusters, reducing the number of LSTM training samples and potentially increasing the variance of predictions. In contrast, in heterogeneous zones, the same threshold may cluster pixels with different temporal profiles, reducing model accuracy. A dynamic thresholding approach can address these issues by setting the correlation cutoff based on local measures of heterogeneity, such as the variance within a moving spatial window of NDI45 or the coefficient of variation in key phenological dates. In practice, areas with low variability may tolerate a higher threshold to preserve cluster homogeneity, while more diverse regions may use a lower threshold to ensure adequate training data. This adaptive method can improve the balance between spatial representativeness and temporal similarity, ultimately increasing the accuracy and robustness of LSTM predictions. Instead of a fixed threshold, a dynamic approach is undoubtedly necessary in a situation where we would like to apply the currently outlined method adaptively to areas with different vegetation characteristics, but its analysis is beyond the scope of our current research.

In the present study, we did not aim to take into account different vegetation types; in the case of a relatively short period of five and a half years, we assumed that similar types of agroecosystems appeared at given locations, which is of course a limitation of our study. The selected 20 × 20 km area on the edge of the Argentine Pampas primarily contains lands under intensive agricultural cultivation (with arable crops with a high leaf area index, e.g., corn, soybeans), and thus the study focuses on arable vegetation patterns. The area does not include significant areas of different vegetation types (e.g., closed forests or other biomes), so the results can be generalized mainly to similar types of agroecosystems. The high-LAI crops have two main consequences for gap-filling performance. First, the seasonal growth curves of these crops exhibit rapid transitions during planting and senescence, which can be challenging for purely smoothing-based methods but can be better captured by the LSTM component of our ensemble. Second, the relative uniformity of crop types within large plots enhances the spatial autocorrelation exploited by correlation-based clustering, improving the training stability of the LSTM and the accuracy of the prediction. However, the phenological phase plays a critical role: in the early vegetative stages, spectral signals change more rapidly and may carry greater uncertainty, while at the canopy tip, NDI45 remains very sensitive to subtle physiological changes, making low MAE reconstruction particularly valuable for stress detection.

In conclusion, the inclusion of land cover information may be useful in the future for interpreting the results and comparing them with other areas, especially if the method is applied to areas with different vegetation types. To improve generalizability, future work should evaluate the KF–LSTM ensemble across a wide range of biome types beyond cropland systems. In closed-canopy forests, the model can be validated using field-measured leaf area index or canopy chlorophyll content, where the challenge lies in capturing subtle physiological changes beneath dense vegetation. In wetlands, the evaluation can focus on the model’s ability to track rapid hydrological changes and seasonal flood cycles, using in situ water level data or SAR-derived inundation maps for validation. For grasslands and savannas, its ability to detect short-lived greening events following combined precipitation can be assessed using high-frequency ground-based phenocam imagery or UAV surveys.

Beyond short-term agricultural decision making, unbiased NDI45 time series have a number of longer-term and cross-sectoral applications. In precision agriculture, the dataset can inform variable input application by identifying zones within an area with different growth vigor or stress during the growing season, even when cloud cover would otherwise obscure critical growth stages. In climate change monitoring, long-term series can be used to detect gradual shifts in vegetation phenology, productivity, and resilience, complementing satellite-based global change assessments. At the policy level, the dataset can support environmental reporting frameworks and Sustainable Development Goal (SDG) indicators on land productivity, food security, and ecosystem health. Additionally, integration with crop yield prediction models, hydrological simulations, and carbon accounting tools could expand its relevance to a variety of stakeholders, from management managers to national agencies.

## 5. Conclusions

This study addressed the challenge of reconstructing continuous, high-resolution Sentinel-2 NDI45 vegetation index time series in cloud-prone regions by developing a variance-weighted Kalman filter–LSTM ensemble. The method combines the deterministic stability of a state–space model with the nonlinear pattern learning capability of deep learning, weighting the output of each model by the inverse error variance. When tested on a 20 × 20 km farmland block in the northern Pampas, the ensemble reduced the mean absolute error by approximately 25–35% (MAE range: 0.009–0.014) and narrowed the 95% confidence interval by 25–40% compared to testing the two models alone. The workflow, implemented in R/Python, is computationally efficient and relies solely on a single optical sensor. Compared to other gap-filling methods, the proposed ensemble offers a distinctive combination of adaptivity, physical interpretability, and explicit pixel-level uncertainty mapping, addressing the lack of methods that combine temporal accuracy, spatial adaptability, and reliability measurements in a single sensor framework.

The current evaluation focused on homogeneous, arable, high-LAI systems; application of the method to forests, wetlands, or heterogeneous grasslands requires biome-specific validation and possibly the inclusion of explicit land cover information. The fixed Pearson correlation threshold (0.75) used for clustering can be dynamically adapted to the local heterogeneity to improve performance.

Overall, the results confirm that the variance-weighted KF-LSTM ensemble improves accuracy, reduces uncertainty, and maintains sensitivity to vegetation changes in the gap-filled NDI45 series, making a transferable and practically valuable contribution to remote sensing and agricultural monitoring. Future research should extend the ensemble to integrate additional predictors, such as SAR reflectance, soil moisture, or meteorological data—and exploring advanced learning methods like Gaussian processes or transformer architectures could further enhance its ability to capture sudden disturbances (e.g., fire, hail, flood). Embedding the process in operational monitoring systems would also provide real-world validation of whether the performance gains lead to measurable economic benefits and better decision making.

## Figures and Tables

**Table 1:** Parameters of the applied KF’s state–space model.

Name	Value
$\theta_{1}$—variance of level disturbance	$\frac{0.07\cdot \bar{x}}{\sqrt{3}}$
$\theta_{2}$—variance of slope disturbance	$\frac{0.07\cdot \bar{x}}{5\sqrt{3}}$
$\theta_{3}$—variance of seasonal disturbance	0.0011
$\theta_{4}$—observation error variance	0.0104

$\bar{x}$—time series mean.

**Table 2:** Parameters of the applied LSTM.

Name	Value
LSTM layer size	32
Batch size	32
Optimizer	Adam
Learning rate	0.005
Loss function	MSE (Mean Squared Error)
Epochs	Max. 128 limited by early stopping
Early stopping patience	5
Input scaling	MinMax scaler

## Data Availability

KF, LSTM, and ensemble model generated layers are available on Zenodo: https://doi.org/10.5281/zenodo.15489840.

## Abbreviations

The following abbreviations are used in this manuscript:

BLUE	Best Linear Unbiased Combination
CNN	Convolutional Neural Networks
EGF-WS	Enhanced Gap Filling and Whittaker Smoothing
EVI	Enhanced Vegetation Index
GRU-D	Gated Recurrent Unit with Decay
HANTS	Harmonic Analysis of Time Series
IDR	Iterative Data Reconstruction
KF	Kalman Filter
KNN	K Nearest Neighbors
LAI	Leaf Area Index
LSTM	Long Short-Term Memory
MAE	Mean Absolute Error
MAPE	Mean Absolute Percentage Error
MCNN	Multiscale Convolutional Network
MICE + MLP	Multiple Imputations by Chained Equations + MultiLayer Perceptron
ML	Machine Learning
NDI45	Normalized Difference Index
NDVI	Normalized Difference Vegetation Index
NDWI	Normalized Difference Water Index
ReCI	Red-edge Chlorophyll Index
RMSE	Root Mean Square Error
RNN	Recurrent Neural Network
SAR	Synthetic Aperture Radar
SAVI	Soil-Adjusted Vegetation Index
SCL	Scene Classification Layer
TFT	Temporal Fusion Transformer
UAV	Unmanned Aerial Vehicle
